# Robust and Efficient Confidence Limits for Phylogenomic Inference of Organismal Relationships

**DOI:** 10.1093/molbev/msaf296

**Published:** 2025-11-18

**Authors:** Sudip Sharma, Sudhir Kumar

**Affiliations:** Institute for Genomics and Evolutionary Medicine, Temple University, Philadelphia, PA 19122, USA; Department of Biology, Temple University, Philadelphia, PA 19122, USA; Institute for Genomics and Evolutionary Medicine, Temple University, Philadelphia, PA 19122, USA; Department of Biology, Temple University, Philadelphia, PA 19122, USA

**Keywords:** phylogenetics, green computing, systematics, little bootstraps

## Abstract

Phylogenomic data are indispensable for establishing reliable relationships needed to build a robust Tree of Life. The superalignment approach concatenates hundreds or thousands of genomic segments, providing a straightforward, computationally efficient, and effective means of inferring phylogenies. However, the standard bootstrap method can produce overly confident support for incorrect inferences based on superalignments. It fails to account for the heterogeneity in phylogenetic signals across the data, which is caused by incomplete lineage sorting (ILS), data errors, and other biological processes. To detect such erroneous inferences, researchers need to produce and deliberate on the concordance of inferences derived from many complex and computationally demanding analyses that require knowledge of data partitions. This study demonstrates that analyzing phylogenomic subsamples with bootstrap upsampling overcomes the overconfidence drawback of the superalignment approach. We found that bootstrapping multiple small, randomly selected site subsets can detect the presence of phylogeny variation signals across the dataset, similar to that detected using data partitions. We present the Net Bootstrap Support (NBS) approach that accounts for this phylogenetic variation in the estimates of bootstrap confidence. *NBS* values showed comparable performance to multispecies coalescent analyses in the presence of ILS and surpassed it for datasets simulated with gene tree estimation errors. NBS analyses of phylogenomic data from rodents, fungi, and carnivorous plants corroborated the performance observed in simulated datasets and even mitigated overconfidence resulting from some data errors. NBS calculations are computationally efficient, with low memory consumption and high computational time savings, making the NBS approach well suited for big data molecular phylogenetics on both desktops and high-performance computing systems.

## Introduction

Analysis of phylogenomic sequence alignments has significantly facilitated the establishment of numerous organismal relationships with high statistical confidence (Gadagkar et al. 2005; Kumar et al. 2012; Song et al. 2012; Kapli et al. 2020; Williams et al. 2020; Sharma and Kumar 2021). The bootstrap support for evolutionary relationships inferred from the analysis of superalignments, consisting of concatenated genes and genomic segment alignments, rapidly converges to 100% for the great majority of inferred clades (Rokas et al. 2003; Gadagkar et al. 2005; Song et al. 2012; Kozlov et al. 2019; Minh et al. 2020; Lanfear and Hahn 2024). This is because increasing the number of genes and genomic segments increases the total number of substitutions available to resolve species relationships and decreases the variance of estimates of branch lengths and other phylogenetic parameters (Philippe et al. 2005; Kumar et al. 2012).

However, superalignment analyses can yield some incorrect relationships with spuriously high bootstrap support (Gadagkar et al. 2005; Shen et al. 2021; Sharma and Kumar 2024). This often arises from overlooking phylogenetic and substitutional pattern variation across genomic loci that negatively impacts phylogenomic inferences (Rokas et al. 2003; Jeffroy et al. 2006; Degnan and Rosenberg 2009; Philippe et al. 2011; Gatesy and Springer 2014; Mirarab et al. 2016). As the number of loci in the dataset increases, the variances of evolutionary estimates become negligible, but the systematic bias resulting from disregarding such variation persists (Kumar et al. 2012). These factors can disproportionately affect the accurate inference of clades with short stem branches, particularly in the presence of incomplete lineage sorting (ILS) (Liu and Yu 2011; Gatesy and Springer 2014; Mirarab et al. 2014; Warnow 2015; Steenwyk et al. 2023; Lanfear and Hahn 2024).

To reduce systematic biases, phylogenetic inference can be performed by fitting different substitution models to individual collections of sites (partitions) using biological and genomic annotations, such as genes, codon positions, or their combinations (Gadagkar et al. 2005; Townsend et al. 2011; McCormack et al. 2012; Simmons and Gatesy 2015; Roycroft et al. 2020; Sharma and Kumar 2022). Such partitions may also be inferred computationally based on the compositional and substitution rate characteristics of sites and genomic segments (Lanfear et al. 2017; Crotty et al. 2020; Minh et al. 2020). In addition to fitting a separate substitution model to each data partition, we need to consider phylogenetic variation across loci. For this reason, multispecies coalescence (MSC) and related approaches are commonly used to combine partition-specific phylogenies to produce species trees (Gadagkar et al. 2005; Liu et al. 2010; Liu and Yu 2011; Mirarab et al. 2014; Edwards 2016; Sharma and Kumar 2024). Nevertheless, analyses involving partitions, including MSC, can suffer from reduced phylogenomic resolution due to many errors in trees inferred from small partitions as well as model misspecification within partitions, overparameterization, and prohibitive computational time and resource requirements (Philippe et al. 2005; Gatesy and Springer 2014; Simmons and Gatesy 2015; Lanfear et al. 2017; Shen et al. 2017, 2021; Molloy and Warnow 2018; Walker et al. 2018; Sharma and Kumar 2024; Ren et al. 2025). A mixture model that allows each site to evolve with its distinct evolutionary pattern can mitigate some systematic biases, albeit at the cost of relatively high computational complexity for large datasets (Crotty et al. 2020; Wong et al. 2024).

**Fig. 1.**
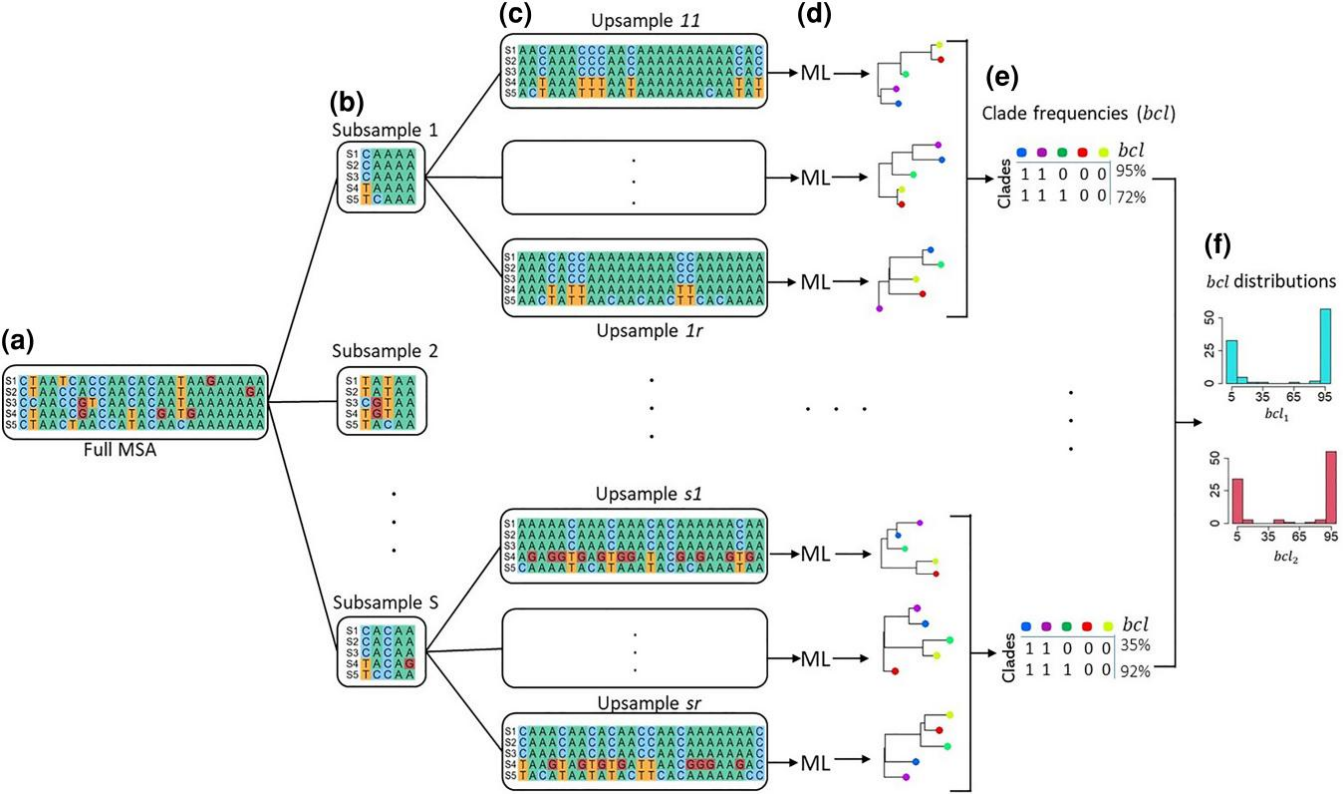
Steps involved in the NBS analysis. a) The full MSA is a superalignment with *L* sites. b) The first step is to draw a subsample of *l* sites without replacement, where l<L. c) This subsample is subjected to bootstrap analysis in which each bootstrap replicate involves generating a replicate MSA from the subsample. The replicate MSA contains *L* sites drawn with replacement from the given subsample of size *l*. This bootstrap replicate is expected to contain multiple copies of each site in the subsample, because l<L. d) A replicate phylogeny is inferred from this replicate MSA using the maximum likelihood or other method of choice. Multiple replicate MSAs and, thus, phylogenies are generated for each subsample. e) A list of inferred clades and their respective frequencies (*bcl* values) in the bootstrap phylogenies produced for each subsample. f) These lists are used to produce a subsample *bcl* distribution for individual clades.

Recently, the bag of little bootstrap approach was introduced for phylogenomics to alleviate the computational burden of bootstrapping the superalignment (Kleiner et al. 2014; Sharma and Kumar 2021). In this approach, bootstrapping is performed on subsamples, each consisting of a small number of sites selected without replacement from the entire superalignment (Fig. 1). For each subsample, bootstrap replicate datasets are created by resampling sites with replacement until each replicate dataset contains the same number of sites as the entire superalignment (Fig. 1a to c). Thus, all the sites in the subsample are included multiple times in the bootstrap replicates generated by this upsampling. The bootstrap replicate datasets produced using Felsenstein's (1985) approach contain, on average, 63% of the distinct site configurations of the entire superalignment (Kleiner et al. 2014; Sharma and Kumar 2021). In contrast, a little bootstrap replicate may contain fewer than 5% of the distinct site configurations of a long superalignment (Sharma and Kumar 2021).

**Fig. 2.**
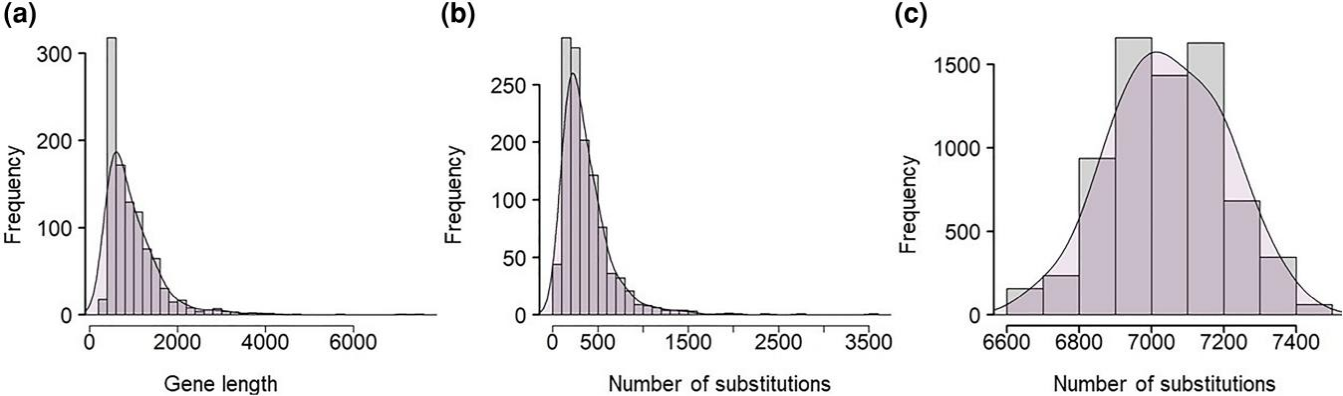
Properties of the phylogenomic dataset containing 1,245 sequence alignments for 37 rodent species. Distributions of a) gene lengths and b) number of nucleotide substitutions for 1,245 sequence alignments obtained from (Roycroft et al. 2020). c) The distribution of the number of nucleotide substitutions in subsamples (1,000). The number of substitutions is the sum of the maximum likelihood estimates of branch lengths for the superalignment ML phylogeny in Fig. 3 multiplied by the number of sites in the subsamples (18,086 bases). Distribution skewness is 3.22, 3.24, and −0.05 for panels a, b, and c, respectively, which are significant for panels a and b (*P* < 0.05).

The random subsampling of sites differs from MSC and other partitioned analyses, as the data partitions are often constructed with consideration for biological and evolutionary factors. Furthermore, these partitions have unequal lengths and numbers of substitutions (Fig. 2). The distribution of sequence length (Fig. 2a) and the estimated number of substitutions (Fig. 2b) is highly skewed for the example phylogenomic dataset, which consists of 1,245 nuclear genes in rodents (Roycroft et al. 2020). Combining phylogenetic inferences from such small and heterogeneous collections of partitions is challenging (Philippe et al. 2005; Townsend et al. 2011; McCormack et al. 2012; Gatesy and Springer 2014; Simmons and Gatesy 2015). In contrast, the little bootstrap framework generates subsamples of equal length, which have a similar expected number of substitutions (Fig. 2c). This uniformity facilitates direct summarization of phylogenetic inferences from little bootstrap replicates (Sharma and Kumar 2021).

Figure 1, depicting a schematic of the little bootstrap analysis, shows that a subsample-wise bootstrap confidence limit (*bcl*) is estimated for clades for each subsample (Fig. 1e). (Sharma and Kumar 2021) showed that Felsenstein's bootstrap support (*FBS*) for a clade is similar to the median of *bcl* values (*fbs*) received across subsamples for the clade of interest: *FBS* ≈ *fbs* = median(*bcl*). However, a few clades with *FBS* ≥ 95% obtained *fbs* < 95% in the analysis of empirical datasets, as shown in Table 1 of Sharma and Kumar (2021). We observed that many subsample *bcl* values were lower than 50% in these situations. This observation led to the hypothesis that phylogenetic variation across sites could be detected from randomly selected subsamples of sites, analogous to the variation reported for biologically annotated data partitions (Lanfear and Hahn 2024; Zhang et al. 2025).

In the following, we present results from our exploration of subsample *bcl* distributions for individual clades in inferred superalignment phylogenies from three empirical datasets. Based on the observed heterogeneity of *bcl* values across subsamples, we have developed the Net Bootstrap Support (*NBS*) metric to account for the overconfidence of *FBS*. We evaluated the performance of NBS analysis by analyzing datasets simulated with varying levels of ILS and gene tree estimation errors (GTEE), as the correct relationships are known for computer-simulated datasets. The *NBS* performance was also compared with that of a multilocus approach, MSC analysis, which is often used to avoid the pitfalls of superalignment analysis.

## Results

### Analysis of Subsample *bcl* Distributions

**Fig. 3.**
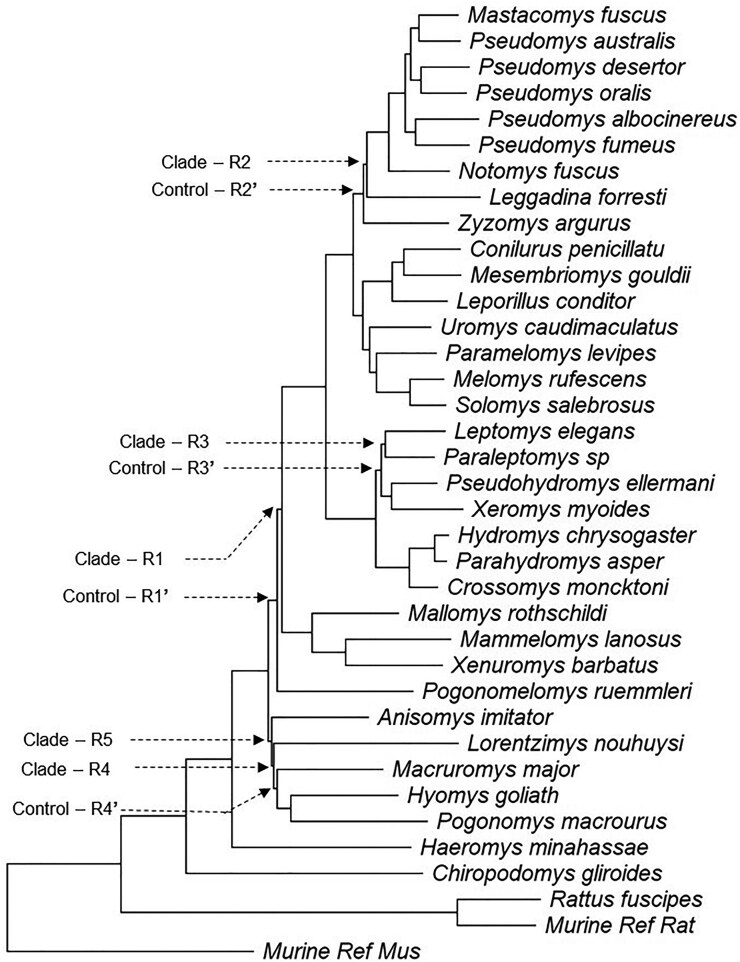
A phylogeny of 37 rodent species. The maximum likelihood tree inferred from the concatenated multiple sequence alignment of 1,245 nuclear coding regions; see Roycroft et al. (2020). *FBS* is greater than 95% for all the clades. Clades exhibiting bimodal *bcl* distributions (R1 to R5) and four adjacent clades (R1′ to R4′) are highlighted.

We begin by presenting results from the analysis of a superalignment containing 1,245 coding regions, with an average length of 970 nucleotides, from 37 rodent species (Roycroft et al. 2020). Its FBS analysis produced a maximum likelihood (ML) phylogeny with ≥95% support for all the clades (Fig. 3). However, four of the inferred clades (R1 to R4) are controversial, as they did not receive support in MSC and other analyses (Roycroft et al. 2020; Shen et al. 2021). This prompted us to conduct a little bootstrap analysis in which 100 subsamples were generated. Each subsample was produced by randomly selecting sites without replacement from the superalignment (Fig. 1). The number of sites sampled was determined automatically using the protocol in Sharma and Kumar (2021) (see Materials and Methods). Bootstrap analysis with upsampling replicates was performed for each subsample, resulting in 100 subsample *bcl* values for each clade in the superalignment ML phylogeny.

**Fig. 4.**
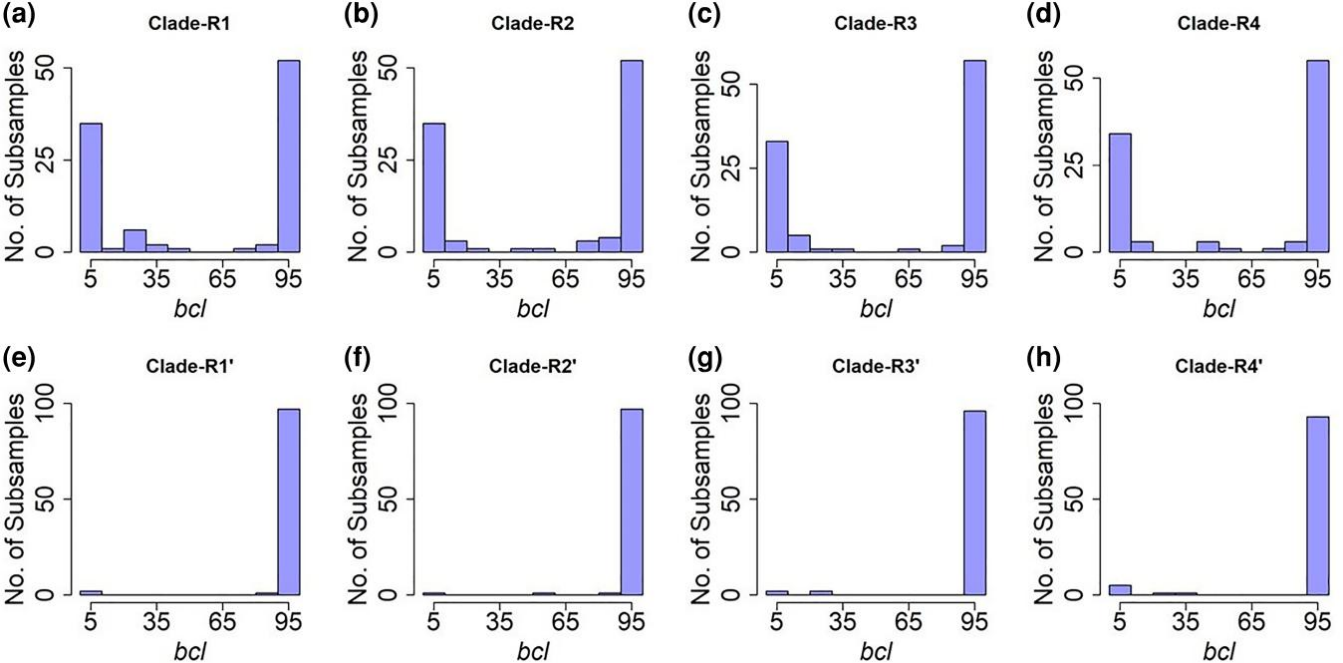
Distribution of subsample *bcl* values for selected clades in the rodent phylogeny. Subsample *bcl* distributions for clades R1 to R4 are shown in panels a) to d), respectively, whereas panels e) to h) show *bcl* distributions for clades R1′ to R4′, which are adjacent to R1 to R4. The test of unimodality (dip.test) of *bcl* distributions for clades R1 to R4 rejected the null hypothesis of unimodality (*P* << 0.05).

Distributions of subsample *bcl* values for clades R1 to R4 exhibited high dispersions, characterized by significant peaks at two tails (Fig. 4a to d). Only ∼44% of the subsamples supported clade R1 (*bcl* ≥ 95%), but more than 30% contradicted it strongly (*bcl* ≤ 5%). The other three clades also exhibited similar patterns, where a large fraction of subsamples opposed the clades inferred using superalignment (Fig. 4b to d). In contrast, subsample *bcl* distributions were unimodal for clades R1′ to R4′ found in the proximity of R1 to R4, respectively (Fig. 4e to h). R1′ to R4′ have relatively short stem branches, similar to R1 to R4, but their *bcl* distributions have a single peak at the right end of the distribution (*bcl* ≥ 95%; Fig. 4e to h).

We performed the maximum deviation test (Hartigan and Hartigan 1985) to assess the unimodality of *bcl* distributions for every clade in the superalignment phylogeny of rodents (see Materials and Methods). It rejected unimodality for clades R1 to R4 as well as another clade, R5, which contains R4 and one other species (see Fig. 3). The kernel density test (Silverman 1981) confirmed the presence of two distinct modes in the subsample *bcl* distributions for clades R1 to R5 (see Materials and Methods). All other clades in the ML tree showed a unimodal *bcl* distribution, with a peak at *bcl* ≥ 95%.

### Analysis of Additional Phylogenomic Datasets

**Fig. 5.**
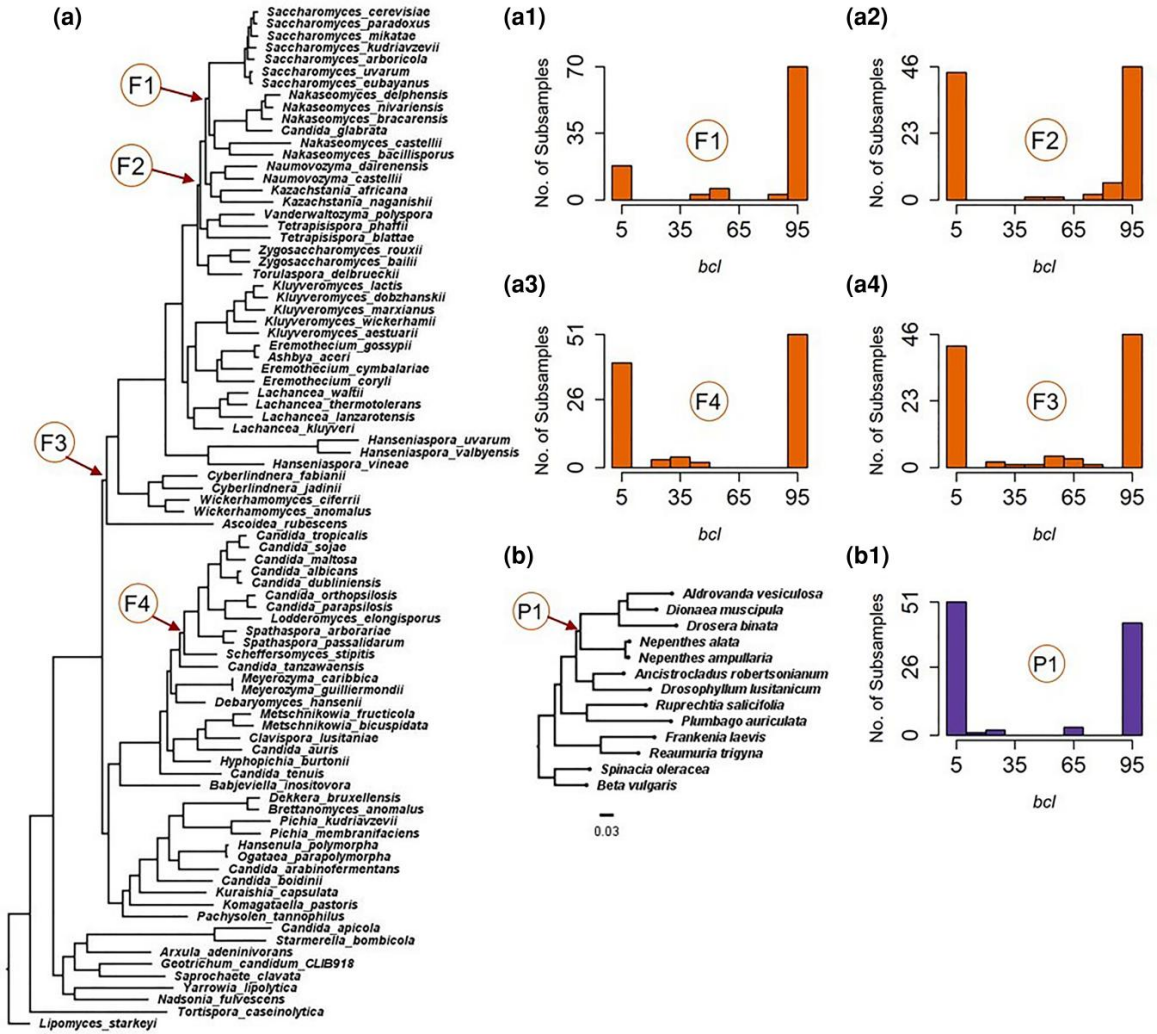
ML phylogenies with subsample *bcl* distributions. a) The ML phylogeny of 86 fungal species was reconstructed using a superalignment of 1,233 nuclear genes. A statistical test identified four clades denoted by F1 to F4 (panels a1) to a4)) where *bcl* values deviated from a unimodal distribution (*P* < 0.05), while *FBS* was greater than 95%. b) The ML phylogeny of 13 carnivorous plant species based on a superalignment of 1,237 genes. The *bcl* distribution for clade P1 (panel b1)) also deviates from a unimodal distribution (*P* << 0.05) even with very high *FBS* (>95%).

To assess the generality of the bimodality trend for the rodent dataset, we analyzed two additional empirical phylogenomic datasets: Fungi (Shen et al. 2016) and Plants (Walker et al. 2017). For the Fungi dataset (1,233 genes), upsampled-bootstrapping of subsamples rejected the unimodality of *bcl* distributions for eight clades in the superalignment ML phylogeny. Four of these clades had received high *FBS* ≥ 95% (Fig. 5a), whereas the other four were not well supported (*FBS* < 59%). For all eight clades, the unimodality test predicted two modes (Fig. 5), so the bimodality of *bcl* distributions appears to be a common characteristic of clades negatively impacted by phylogeny variation across sites in a phylogenomic dataset.

A similar pattern was observed for ML phylogeny of carnivorous plants (Fig. 5b), inferred from the superalignment of 1,237 genes. The unimodality of the *bcl* distribution for only 1 clade out of 10 was rejected, where two modes were detected (Fig. 5b1). This clade in the plant phylogeny and four clades of Fungal phylogeny (all with *FBS* ≥ 95%) have been controversial (Shen et al. 2017; Walker et al. 2018; Sharma and Kumar 2024). These results are noteworthy because the subsample *bcl* distributions are derived from data that contain random samples of sites, rather than being delineated based on knowledge of biological attributes. Also, the bimodality of these *bcl* distributions appears to be a hallmark of extensive phylogenetic variation across the genome.

### Revising the Bootstrap Support: Net Bootstrap Support

Bimodal subsample *bcl* distributions, particularly for well supported but controversial clades (i.e. high *FBS*), provided a way to estimate the overconfidence (*OC*) of *FBS* values. For a clade, *OC* is the average signed difference between individual subsample *bcl* values and the superalignment *FBS*. It can be written as:


(1) $$OC\;=\;\frac{1}{S}\sum_{i=\;1}^{S}\;(FBS_{\;}-bcl_{i})$$

Here, *S* is the total number of subsamples analyzed. Because of the signed term in Equation 1, *OC* will be 0 when the *bcl* distribution is balanced and symmetrical around *FBS*.

We estimate the *NBS* for a clade as:


(2) $$NBS_{\;}\;=\;FBS_{\;}-OC_{\;}$$

This can be expressed as:


(3) $$NBS_{\;}\;=\;FBS_{\;}-\frac{1}{S}\sum_{i\;=\;1}^{S}\;(FBS_{\;}-bcl_{i})$$

Equation 3 simplifies to,


(4) $$NBS_{\;}\;=\;\frac{1}{S}\sum_{i\;=\;1}^{S}\;(bcl_{i})\;\;\;=\;mean_{\;}\{bcl\}$$

Therefore, the mean of the *bcl* distribution is the bootstrap support when excluding the effect of phylogenetic heterogeneity across data. Importantly, the use of the mean to estimate *NBS* is the algebraic outcome of the definition of *NBS* as shown by Equations (1)–(4). It represents the net frequency of clade support across the data, even when the *bcl* distribution is not unimodal or symmetrical.

*NBS* can be calculated directly from the little bootstraps approach using Equation (4), obviating the need for a time-consuming standard bootstrap analysis of the entire superalignment. Because the median of the subsample *bcl* distribution approximates *FBS* well (Sharma and Kumar 2021), one can obtain both *NBS* and *FBS* estimates from the same subsample *bcl* distribution without needing to conduct a standard bootstrap analysis of the entire superalignment.

### Performance of NBS Analysis for Simulated Data Collections

#### Analysis of Datasets With ILS

We first analyzed datasets that evolved with ILS, which is known to cause high *FBS* values for incorrect relationships in superalignment phylogenies (Simmons and Gatesy 2015; Warnow 2015). We selected an independently generated dataset previously used to study the impact of low, medium, and high degrees of ILS on phylogenomic inference (Mirarab et al. 2014). This dataset consists of 100 gene sequence alignments, each 1,600 bp in length, from 37 species; see Fig. S1 for the model phylogeny and Materials and Methods for details. We analyzed 10 collections of alignment for low, medium, and high ILS situations.

**Fig. 6.**
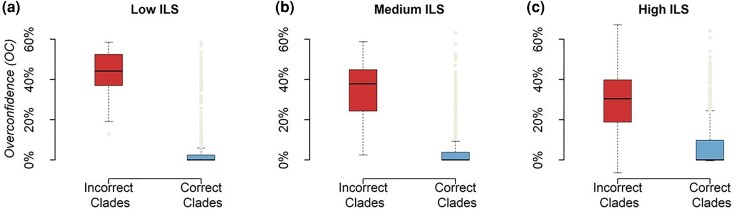
Comparison of *OC* values for correct and incorrect clades for datasets simulated with ILS. Box plots of *OC* values are shown for incorrect clades (left) and correct clades (right) in superalingment ML phylogenies derived from datasets simulated with a) low, b) medium, and c) high levels of ILS. Boxes represent the interquartile range (IQR). The horizontal line within each box denotes the median, and whiskers extend to 1.5 times the IQR. Individual dots represent outliers. For all ILS conditions, the median *OC* for incorrect clades is significantly higher than for correct clades (Wilcoxon signed-rank test, *P* << 0.05).

**Fig. 7.**
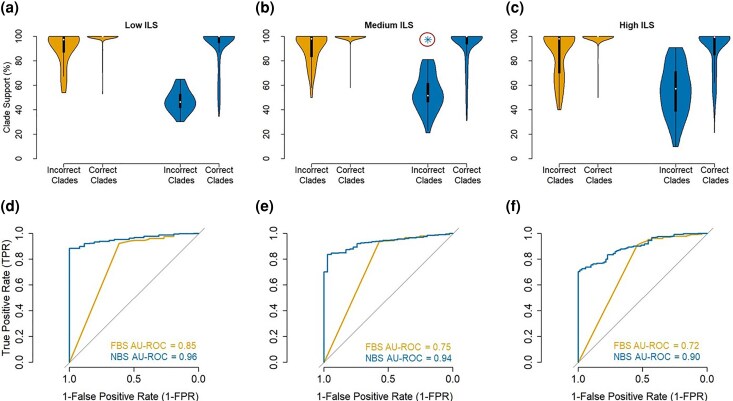
Comparison of *NBS* and *FBS* values for correct and incorrect clades for datasets simulated with ILS. Violin plots show distributions of *NBS* (left) and *FBS* (right) for correct and incorrect clades in superaligment ML phylogenies derived from datasets simulated with a) low, b) medium, and c) high levels of ILS. The circle in panel b) highlights an incorrect clade supported by *NBS* ≥ 95%; the Type I error threshold was 5%. Panels d) to f) show receiver operating characteristic (ROC) curves for *NBS* and *FBS* values in distinguishing correct versus incorrect clades across the same ILS conditions. The area under each ROC curve (AU-ROC) is reported, with *NBS* consistently showing higher AU-ROC values than *FBS*, indicating superior discriminative power. The diagonal line represents the expected performance of a random classifier.

Superalignment ML phylogenies exhibited 8%, 10%, and 21% incorrect clades for the low, medium, and high ILS datasets, respectively. Incorrect clades tended to have shorter stem branches, with the average lengths for correct clades being approximately three times longer than those for incorrect clades. We expected high *OC*s for these incorrectly classified clades. Indeed, their median *OC*s ranged from 30.4% to 44.2% for high, medium, and low-level ILS datasets (Fig. 6a to c). In contrast, *OC* for correct clades was minuscule (≤ 0.5%, Fig. 6a to c). These differences between correct and incorrect clades were statistically significant (*P* < 0.01) in nonparametric Wilcoxon signed-rank tests (see Materials and Methods). Consequently, *NBS* was relatively low for almost all the incorrect clades that received *FBS* ≥ 95% (Fig. 7a to c). The false positive rate (*FPR*) for *NBS* was less than 1% at a 95% cutoff (Fig. 7d to f). Receiver operating characteristic (ROC) curves show that the reduction of *FPR* was achieved without sacrificing the true positive rate (*TPR*; Fig. 7d to f), making *NBS* an excellent choice to replace *FBS*.

We also compared the AU-ROC for *NBS* ROC curves with local posterior probabilities (*LPP*) from MSC analysis. The area under each ROC curves (AU-ROCs) for high, medium, and low ILS datasets for *NBS* were similar to *LPP* values from MSC analysis, ranging from 0.93 to 0.96. Therefore, NBS analysis overcomes the bias of *FBS*, similar to that afforded by the MSC analysis. However, unlike MSC analysis, NBS analysis does not need the knowledge of partitions.

#### Analysis of Datasets with Gene Tree Estimation Errors

**Fig. 8.**
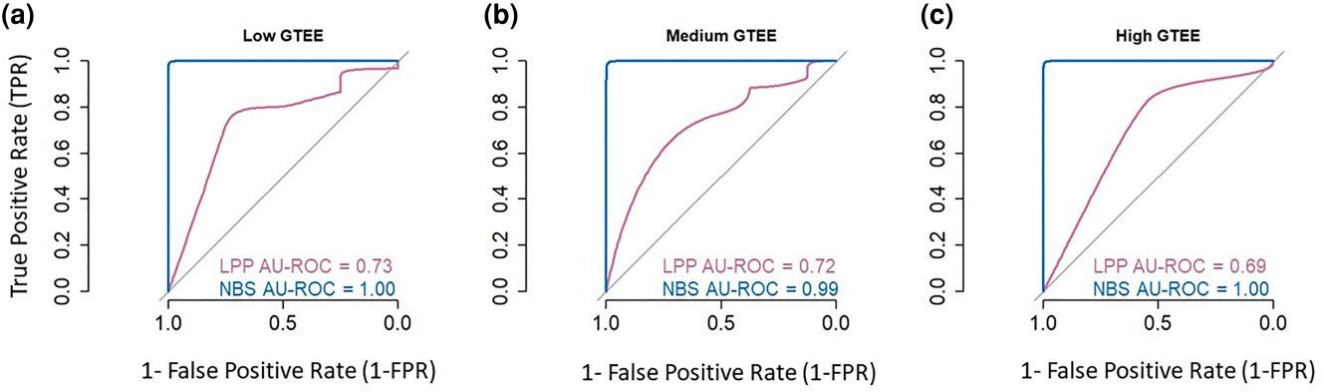
Comparison of *NBS* and *LPP* for datasets simulated with GTEE. ROC curves for *NBS* and MSC *LPP* values in identifying correct and incorrect clades derived from datasets simulated with a) low, b) medium, and c) high levels of GTEE. Area under the ROC curve (AU-ROC) is displayed with each ROC curve. The area under the ROC curve (AU-ROC) is reported for each method in each condition. Across all levels of GTEE, *NBS* consistently exhibits higher AU-ROC values than MSC *LPP*, indicating its superior ability to maintain high support for correctly inferred clades, even in the presence of extensive gene tree uncertainty. These results demonstrate that *NBS* is more robust to GTEE than MSC-based support values, providing a more reliable measure of clade confidence when GTEE occurs.

We analyzed another simulated data collection to investigate the relative performance of NBS and MSC analyses for datasets exhibiting large GTEE (see Materials and Methods). These datasets were simulated with low, medium, and high levels of GTEE (see Materials and Methods). Both *FBS* and *NBS* values were ≥95% for all correct relationships, except one clade that received a *NBS* value of 90%. This clade had the shortest stem branch in the phylogeny. Therefore, the overall *TPR* for *NBS* was >98% (AU-ROC = 0.99 to 1.00, Fig. 8a to c) at a 95% cutoff level. In contrast, *TPR* for *LPP* in MSC analysis was only 72% at a 95% cutoff level (AU-ROC = 0.69 to 0.73, Fig. 8a to c), resulting in a lower overall accuracy compared to *NBS* values for all levels of GTEE analyzed. These results are consistent with the known drawback of MSC analyses, which are influenced by the accuracy and resolution of individual partition phylogenies (Salichos and Rokas 2013; Simmons and Gatesy 2015; Molloy and Warnow 2018).

#### Comparison with the Double Bootstrap Analysis

We compared the performance of NBS analysis with that of the double bootstrap method, which is intended to correct the bias of standard bootstrap analysis (Beran 1988). In this method, the resampled bootstrap dataset (outer loop) undergoes an additional round of resampling (inner loop) before the estimator (e.g. phylogeny) is computed. Because ∼63% of the sites are represented in each round of resampling, the representation of the sites in the superalignment is expected to be reduced to ∼39% (0.63 × 0.63). This proportion is far too large to capture most of the variation in phylogenetic signal across the superalignment. Indeed, the AU-ROC of the double bootstrap support (*DBS*) was only slightly better than *FBS* for datasets simulated with low (0.91 vs 0.85), medium (0.84 vs 0.75), and high (0.77 vs 0.72) levels of ILS, but much lower than *NBS* (low: 0.96, medium: 0.94, and high: 0.90). In the analysis of empirical datasets, *DBS* estimates were greater than or equal to 95% for all the controversial clades, except F2 for which *DBS* was reduced to 90% from 100% for *FBS*. For F2, *NBS* was much lower (53%). Therefore, *DBS* does not overcome the shortcomings of *FBS*, as does the *NBS*.

## Discussion

We have presented a novel approach to incorporating the uncertainty in measuring the statistical support for organismal relationships inferred using superalignments. It utilizes the little bootstrap framework to determine the heterogeneity of clade phylogenetic support using small subsamples of randomly selected sites. In investigations involving empirical datasets, the revised statistical support (*NBS*) performed well in the presence of three significant types of adversities that impact the performance of phylogenomic inference.

One is the adverse impact of data errors, which can be attributed to overly influential loci (Shen et al. 2017; Sharma and Kumar 2024). For example, *NBS* (46%) for the plant clade P1 was much lower than *FBS* (100%) (Fig. S2). This result is consistent with reports that P1 is not robust to the exclusion of just two out of 1,237 genes (Walker et al. 2018). These two genes are suspected to be paralogs that were inadvertently retained during the data curation process (Walker et al. 2017, 2018). NBS analysis was not misled by these data errors, which was also the case for the MSC analysis, as it did not support clade P1 (*LPP* = 53%). A similar situation exists for the clade F3 in the Fungi phylogeny (Fig. 5a), which was not robust to the exclusion of a single gene out of 1,233 (Shen et al. 2017; Sharma and Kumar 2024). The gene tree of that influential gene exhibited a spuriously long branch in the middle of the phylogeny and is suspected to be a hidden paralog or misidentified ortholog (Shen et al. 2017; Sharma and Kumar 2024). The *NBS* value for clade F3 was low (56%), despite an *FBS* greater than 95%. Therefore, *NBS* can overcome the shortcomings of *FBS* when problematic genes produce misleading *FBS*.

The presence of ILS poses the second type of challenge for the *FBS* analysis of superalignment. In the analysis of computer-simulated datasets, little bootstrapping successfully addressed this shortcoming and produced accurate clade supports that were similar to those of MSC analyses. This is also confirmed in empirical data analyses. For example, the clustering of rodent taxa in clade R1 of the superalignment ML phylogeny was detected to be spurious by NBS analysis, which has been suggested to be due to ILS (Shen et al. 2017, 2021; Roycroft et al. 2020) (Fig. S4). Other clades with low *NBS* values received quartet support values of less than 38% (Roycroft et al. 2020; Shen et al. 2021), which is not much higher than 33.33% expected for a trichotomy. Also, MSC analyses did not support F1, F2, and F4 clades in the superalignment ML phylogeny (Fig. S3). Therefore, NBS analysis overcomes spurious support from traditional bootstrap analysis of superalignments in these situations.

Third, NBS analysis performed well for datasets with GTEE, recovering all correct species relationships with a high support in the analysis of simulated datasets (Fig. 8). MSC *LPP* did not perform well in producing clade support, as the MSC approach primarily focuses on handling phylogenetic variation across partitions arising from ILS (Liu et al. 2010; Mirarab et al. 2021; Wong et al. 2024). However, the phylogenetic variation observed in the simulated gene trees was predominantly caused by GTEE, stemming from a limited number of substitutions (1 to 2) per site in each sequence alignment. It is well known that MSC can perform poorly in estimating species phylogeny and clade support when phylogenetic variation is due to GTEE (Simmons and Gatesy 2015; Shen et al. 2021). Unlike MSC, each little bootstrap replicate dataset is expected to contain as many substitutions as the entire superalignment, so *NBS* can be high for clade inferences with even short internal branches when GTEE is high (Fig. S5). Therefore, NBS can achieve higher accuracies than FBS analysis of superalignments in many instances and MSC analysis in some cases.

These gains in the accuracy of the clade support offered by NBS analysis are accompanied by large savings in computational time and memory. This occurs because NBS analysis uses the little bootstraps approach, where each subsample and, thus, bootstrap replicate dataset contains only a minuscule fraction of distinct site patterns compared to the majority (∼63%) expected to be included in the standard bootstrap replicate dataset. In the empirical analyses presented, NBS analysis required sampling only a small proportion of sites (1.4% to 2.1% in both empirical and simulated datasets), resulting in up to 100-fold reductions in memory usage and 10-fold speedups compared to *FBS* calculations (see Table S1). Due to the small memory footprint, bootstrap replicates for NBS can be executed in parallel on personal desktops, thereby further reducing the wall time required to complete phylogenomic analyses. Therefore, the use of NBS will not only address the statistical limitations but also alleviate the substantial computational burden of standard bootstrap analysis on large phylogenomic datasets.

## Materials and Methods

### Datasets Analyzed

#### Empirical Datasets

We have obtained three empirical datasets from previously published phylogenomic studies of three groups of organisms: rodents (Roycroft et al. 2020), fungi (Shen et al. 2016, 2017), and plants (Walker et al. 2017) (Table 1). They were selected because the ML phylogeny inferred from the concatenated superalignment analysis received very high *FBS*, some of which was not reproduced in other analyses.

**Table 1.** Phylogenomic datasets analyzed

Dataset	Data type	Number of species	Number of genes	Gene length (Minimum to Maximum)	Superalignment length
Rodent (R)	DNA	37	1,245	249 to 7,413	1,207,638
Plants (P)	DNA	13	1,237	336 to 15,743	2,117,583
Fungi (F)	Amino acid	86	1,233	167 to 4,854	609,772

#### Simulated Datasets

We gathered simulated gene sequence alignments from a previously published article (Mirarab et al. 2014) that explored various levels (low, medium, and high) of ILS on inferred species phylogeny. We generated 10 datasets, each with 100 gene sequences sampled from 4,000 simulated gene sequence alignments (Mirarab et al. 2014). Each gene sequence contained 1,600 sites and was simulated using ILS, modeled through a MSC approach. The model species tree, containing 37 mammalian species, was used for simulation (Fig. S1). Consequently, each dataset contains a total of 160,000 sites (Mirarab et al. 2014). The simulated GTEE datasets were obtained from Shen et al. (2021). Three gene trees with identical topologies to the model species phylogeny were generated, but with branch lengths scaled by 0.1, 0.07, and 0.05, respectively, to simulate low, medium, and high GTEE. For each GTEE level, 1,000 gene sequence alignments were simulated.

#### Species Tree Inference

We inferred species phylogenies using superalignment analysis and partitioned MSC approaches. The ML approach was employed to infer the superalignment phylogeny using IQ-TREE 2 (Minh et al. 2020). Similarly, we also inferred gene trees for MSC analysis using the ML approach in IQ-TREE2. The MSC phylogeny was inferred from the collection of individual gene trees using ASTRAL (Mirarab et al. 2014).

For all nucleotide sequences, we used the generalized time-reversible (GTR) model and the gamma-distributed rate of substitutions with four categories (G4) as the substitution model. For amino acid sequence alignments, we employed the LG model with gamma-distributed rates of substitutions (G4) as the substitution model (Le and Gascuel 2008).

#### Calculation of *FBS* and *LPP* Support Values

*FBS* for the superalignment phylogeny was calculated using 100 bootstrap replicates in IQ-TREE 2 for the rodents and plants data. *FBS* values for the Fungi phylogeny are from Shen et al. (2017), who reported taking ∼70 wall clock hours to perform the ML tree inference from a single standard bootstrap replicate (Table S1). The *LPP* for clades in species trees from the MSC analysis were estimated using ASTRAL (Mirarab et al. 2014).

#### Calculation of *NBS* Values

We developed a computational pipeline for estimating *NBS*. This pipeline determines the number of sites to subsample and the number of subsamples to analyze. It produces *NBS* values for clades in the given phylogeny and a bootstrap consensus phylogeny, along with *NBS* when no phylogeny is provided. The number of sites subsampled is selected using the approach presented in Sharma and Kumar (2021). For empirical datasets, the number of sites included in each subsample is given in Table S1. For simulated datasets, the number of sites sampled in subsamples averaged 1.4% to 1.6%. For initial exploration of the *bcl* distributions using simulated and empirical superalignments, a total of 10,000 subsample bootstrap replicates were generated (100 subsamples with 100 upsample replicates each). The ML phylogeny for each subsample bootstrap replicate was inferred using IQ-TREE 2.

For general NBS analysis, 5 subsamples were generated along with 20 upsampled replicates per subsample to ensure a 5% resolution in the estimated bootstrap values. The number of upsampled replicates was kept fixed, and the number of subsamples was increased adaptively until the root mean squared deviation (RMSD) of *NBS* values between successive iterations stabilized. RMSD was calculated using the formula:


$$RMSD_{S\;}=\sqrt{\frac{1}{C}\left\{\overset{C}{\underset{i\;=\;1}{\sum }}\;{\left(NBS_{i}^{j}-NBS_{i}^{\;j-1}\right)}^{2}\right\}}$$

where *C* is the total number of clades in the phylogeny, $NBS_{i}^{j}$ and $NBS_{i}^{\;j-1}$ represent the *NBS* values for the *i*th clade from *j*th and *(j − 1)*th subsamples. The process continued until the RMSD fell below 0.05. We implemented this pipeline as an *R* function, which produces *NBS* estimates and the consensus *NBS* tree. To validate results, one can optionally double the subsample size and repeat the analysis to check for stability in the clade support inference.

#### Handling Data-poor Taxa in Phylogenomic Alignments

Three empirical datasets analyzed above had excellent data coverage across taxa, as the maximum fraction of sites with missing and ambiguous bases for any taxon (*mf*) was substantial but modest for the fungus (44.39%), rodent (2.95%), and plant (29.02%) datasets. However, *mf* can be large in some phylogenomic datasets. For instance, *mf* was 95.90% for a phylogenomic dataset of 39 birds (Yonezawa et al. 2017). For one species, only 2,533 sites contained a nucleotide base in the superalignment, which consisted of 61,794 sites. In this dataset, more than 50% sites were missing data for a majority of taxa. For such datasets, the automated selection of the number of sites in subsamples, as presented in Sharma and Kumar (2021), suggested sampling 6,408 sites from the superalignment, which resulted in the sampling of only 252 to 281 valid bases for the taxon for which only 2,533 sites had valid bases. While the *NBS* estimation remains feasible, one must be cautious when including such data in superalignments. We recommend removing taxa with extreme data sparsity (e.g. *mf* ≥ 95%) prior to NBS analyses. In fact, our *NBS* estimation pipeline issues a warning if a taxon contains more than 50% missing bases or alignment gaps, consistent with like practice in IQTREE-2 and other software. In any case, if zero sites are sampled for a subsample, then the pipeline skips the subsample with a warning.

#### Test of Unimodality and the Wilcoxon Signed-rank Test

We performed Hartigan's Dip test using the “*dip.test*” function from the R package *dip test* (Hartigan and Hartigan 1985). The null hypothesis for this was that the *bcl* values for a clade have a unimodal distribution. The statistical test was assessed using a 5% level of significance (*α* = 0.05), and the *P*-value was calculated using a Monte Carlo test method with a default number of replicates (2,000). Next, we test for a statistically significant number of modes of at least two or more using the Silverman test, which can be performed using the “*silverman test*” function from the R package *silverman test* (Silverman 1981).

A nonparametric Wilcoxon signed-rank test (Bauer 1972) was performed to assess the statistical significance of the difference in *OC* or clade supports between correct and incorrect clades. A two-sided alternative hypothesis was considered to account for potential differences in either direction. The test was implemented in R using the function “*wilcox.test*” from the package “*stats.*”

## Supplementary Material

msaf296_Supplementary_Data

## Data Availability

All datasets used in this article were obtained from published articles and shared in a Figshare repository: https://figshare.com/s/16d110cc419e4760ee33. The NBS analysis pipeline is available in a GitHub repository https://github.com/ssharma2712/Net_Bootstrap_Support_NBS.
