# Supplementary material for: Robust and Efficient Confidence Limits for Phylogenomic Inference of Organismal Relationships
Source: Mol Biol Evol. 2025 Nov 18;42(12):msaf296. doi: 10.1093/molbev/msaf296 (PMC12665395; doi:10.1093/molbev/msaf296)
Supplement: msaf296_Supplementary_Data [file msaf296_supplementary_data.pdf]

Supplementary Table

Dataset (Data Type)	PSU analysis			Memory per replicate (GB)			Time/Reps (Hours)		
	Subsample Size (sites)	% sites used	Subsamples and Replicates	*FBS*	*NBS*	Fold-Saving	*FBS*	*NBS*	Fold Saving
**Rodent (DNA)**	18,086	1.49%	6 x 20	0.32	0.02	21	0.25	0.05	5
**Fungi (AA)**	11,210	1.83%	7 x 20	19.07	0.18	106	70.5	6.57	10
**Plant (DNA)**	26,797	2.06%	6 x 20	0.51	0.02	28	0.63	0.10	6

Supplementary Table 1: Computational time and memory requirements for ML phylogeny inference using a single replicate from the NBS and FBS estimation in empirical datasets.

Supplementary Figure S1

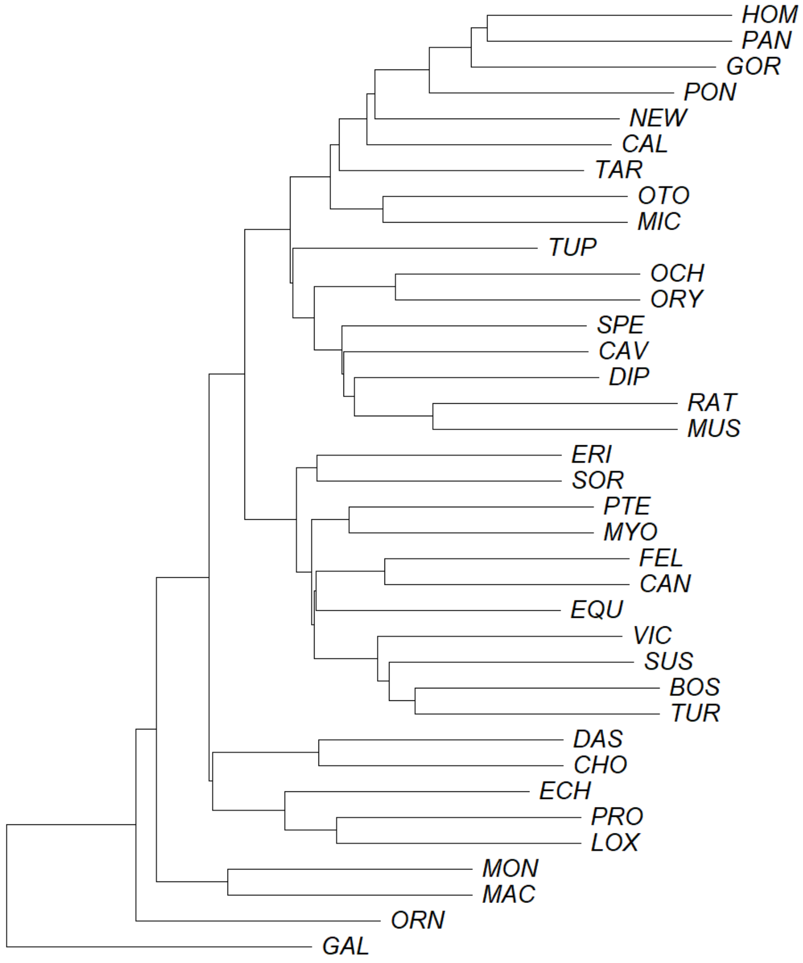

Supplementary Figure S1. The model species tree used for simulating datasets with ILS. This tree is gathered from Mirarab et al. (2014).

Supplementary Figure S2

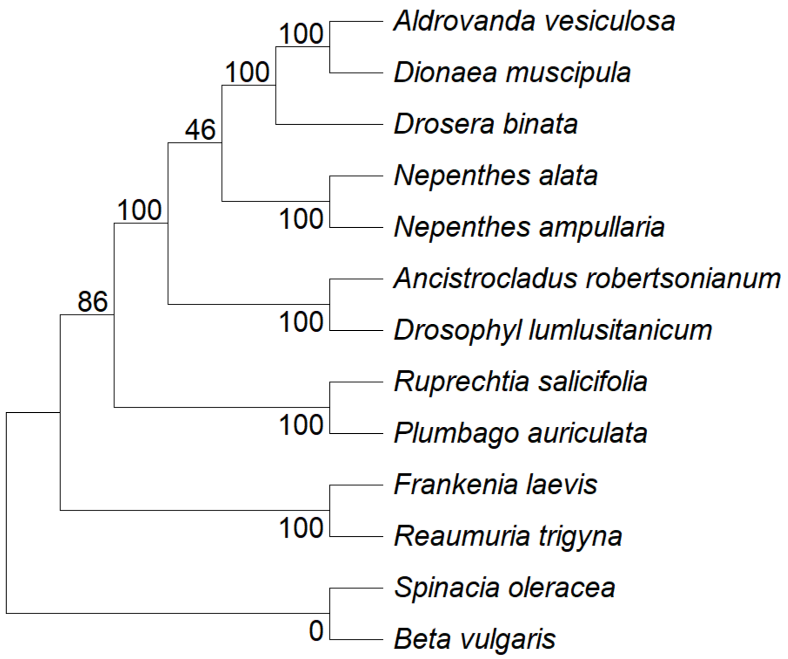

Supplementary Figure S2. The carnivorous plant phylogeny inferred from the Maximum Likelihood analysis of CSA. *NBS* for clades less than 95% are displayed on the phylogeny. The clade P1 received the lowest *NBS*, 46%.

Supplementary Figure S3

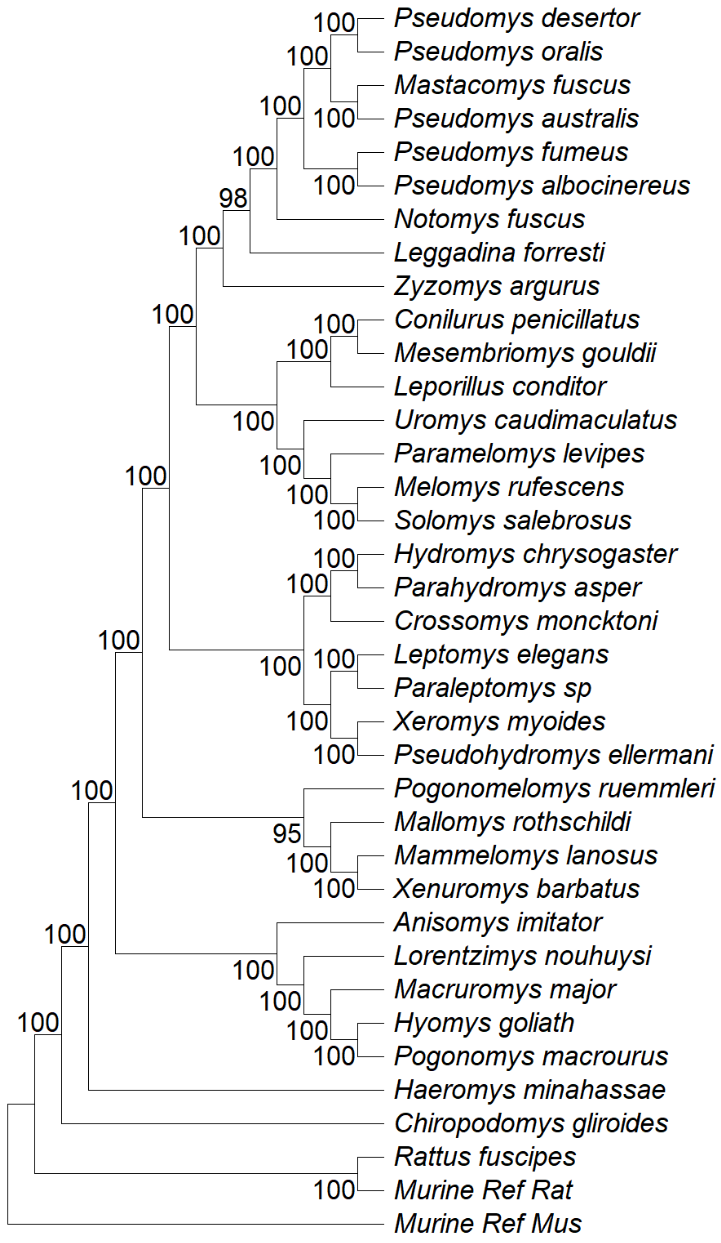

Supplementary Figure S3. The Fungi phylogeny inferred from the MSC analysis using ASTRAL. Clade supports represent the *LPP* estimated using ASTRAL. The phylogeny was collected and reproduced from Shen et al. (2017).

Supplementary Figure S4

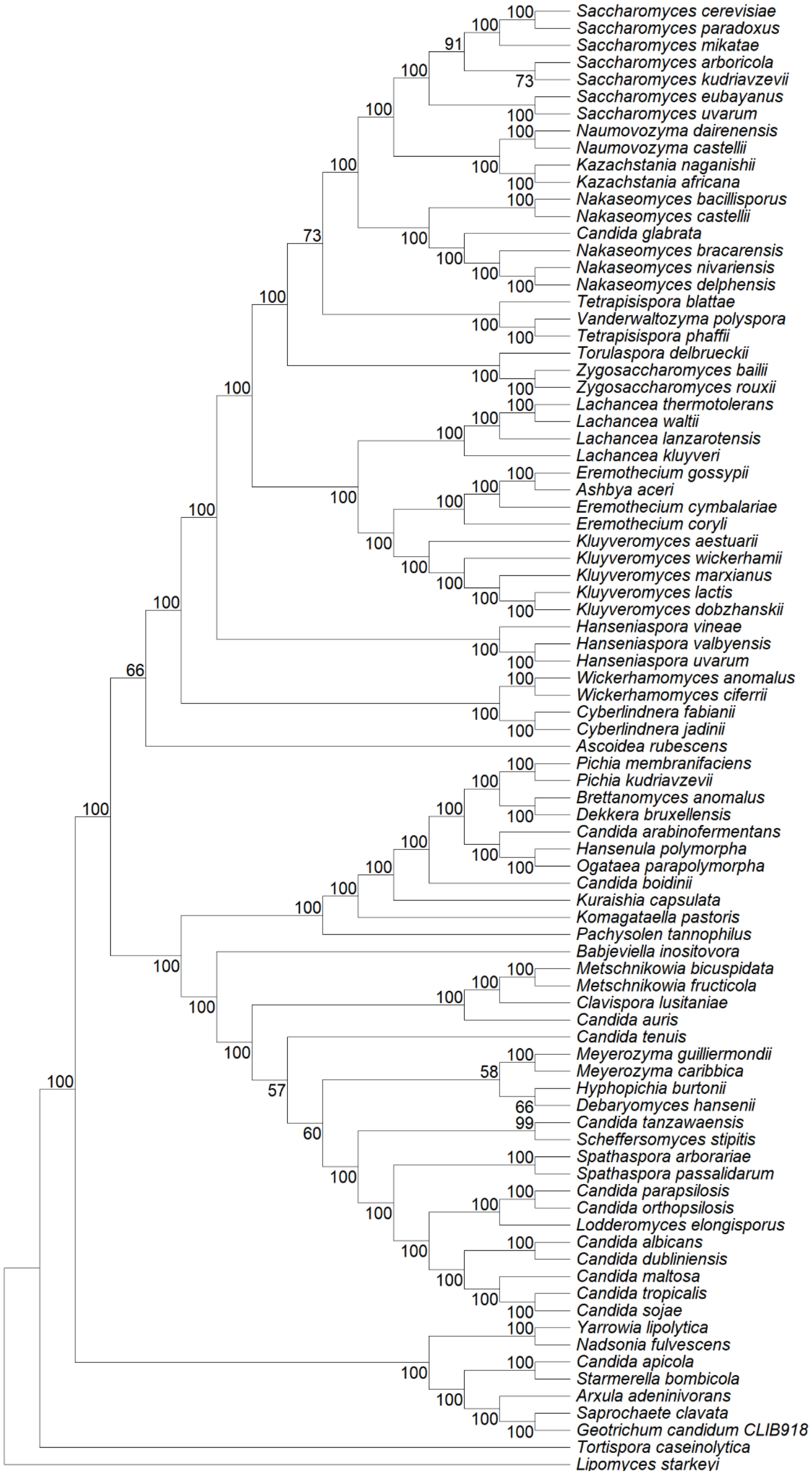

Supplementary Figure S4. Rodent phylogeny inferred using the MSC approach with ASTRAL and retrieved from Shen et al. (2021). Clade support values represent local posterior probabilities estimated by ASTRAL.

Supplementary Figure S5

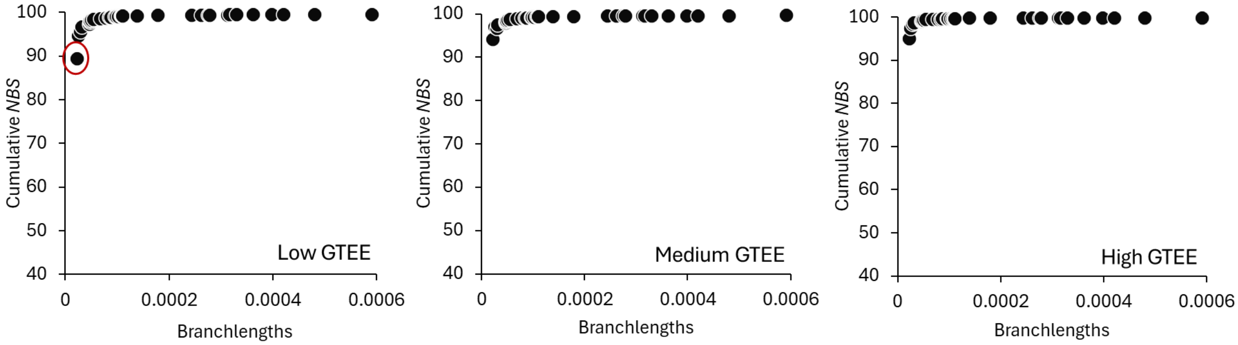

Supplementary Figure S5. Relationship between cumulative average *NBS* and branch lengths of the inferred phylogeny. A low *NBS* value (89.5%, red circle) was observed for the shortest branch in the inferred phylogeny from datasets simulated under low GTEE conditions.
